# Reading “Sun” and Looking Up: The Influence of Language on Saccadic Eye Movements in the Vertical Dimension

**DOI:** 10.1371/journal.pone.0056872

**Published:** 2013-02-27

**Authors:** Carolin Dudschig, Jan Souman, Martin Lachmair, Irmgard de la Vega, Barbara Kaup

**Affiliations:** Department of Psychology, University of Tübingen, Tübingen, Germany; University of Muenster, Germany

## Abstract

Traditionally, language processing has been attributed to a separate system in the brain, which supposedly works in an abstract propositional manner. However, there is increasing evidence suggesting that language processing is strongly interrelated with sensorimotor processing. Evidence for such an interrelation is typically drawn from interactions between language and perception or action. In the current study, the effect of words that refer to entities in the world with a typical location (e.g., *sun*, *worm*) on the planning of saccadic eye movements was investigated. Participants had to perform a lexical decision task on visually presented words and non-words. They responded by moving their eyes to a target in an upper (lower) screen position for a word (non-word) or vice versa. Eye movements were faster to locations compatible with the word's referent in the real world. These results provide evidence for the importance of linguistic stimuli in directing eye movements, even if the words do not directly transfer directional information.

## Introduction

The vast amount of input received by the human visual system at any moment exceeds the brain's capacity to integrate information into conscious experiences. Thus, selective attention and filtering is required in order for incoming information to be effectively processed [1]. Language can serve as an important cue for shifting one's attention to important aspects in the world. For example, we know that processing words such as *up*, *down*, *left* and *right* results in attention shifts to compatible locations [2], [3]. Interestingly, experiential-simulation (embodied) models of language understanding propose interactions between language and other cognitive systems, such as attention, perception and action [4], [5]. For example, we often encounter the word *airplane* in a situation where someone points to an airplane in the sky, thus making us look upwards. According to an experiential-simulation approach of language understanding, these experiential traces (e.g., looking upwards) become reactivated when later accessing the meaning of the word *airplane* [6]. Despite converging evidence for a close relationship between the linguistic system and other cognitive systems, the automatic impact of linguistic stimuli on saccadic eye movements is still to be shown. In the current study, we aim to analyze the influence of words referring to entities in the world with a typical location in the vertical dimension (e.g. *sun* vs. *worm*) on saccadic eye movements in the vertical dimension.

The relationship between language and visual attention was first studied using the so-called visual world paradigm, where participants process linguistic input while exploring visually presented object arrays. Typical findings show that the way participants view visual scenes reflects the way in which sentences are disambiguated [8]. For example, Altmann and Kamide [9] showed that eye movements are directed to objects prior to the actual object's first mention in the sentence when the developing context limits the subsequent options available. Similarly, visual referential scenes strongly guide sentence processing in a manner which even eliminates strong syntactic preferences such as typically observed in garden-path sentences [10]. Indeed, these findings suggest a close link between complex language processing and eye movements, whereby the eyes are automatically directed to relevant parts in the visual field. Importantly, in these paradigms, the visual scenes typically provide additional information that might be actively accessed and integrated into the discourse model.

Additional evidence for the close relationship between language and visual space has been drawn from attentional cueing paradigms in the tradition of Posner's work on attentional orienting [11]; specifically, the automatic influence of single word processing on involuntary attentional shifts triggered by directional words (*above*, *below*, *left* or *right*) was investigated (e.g., [3]). Hommel, Pratt, Colzato and Godijn [2] showed that task-irrelevant directional words such as *up*, *down*, *left* and *right* result in reflexive shifts of visual attention towards the compatible location. In their experiments, centrally presented words were followed by a target which randomly appeared in the top, bottom, left or right position. Importantly, the words' meaning did not transfer any beneficial information for task performance. However, participants were faster in processing a target in a congruent location (e.g., the word *up* facilitated target processing in an upper screen location) than in an incongruent location. Hommel et al. replicated these findings in several experiments implementing different experimental tasks (e.g., target detection vs. target discrimination task). Additionally, Hodgson et al. showed that directional words also facilitate eye movements in compatible directions [12]. Here, directional words were presented in a Stroop-like paradigm [13]. The words were presented centrally in different font colors along with colored squares at each side of the screen (e.g., top = green, bottom = blue, left = red & right = yellow). Participants had to respond by moving their eyes to the square that matched the font color. Increased saccadic latency, in addition to an increased error rate, was observed for incompatible trials (e.g., word = *up*, but the word's color demands a downwards saccade). These findings are important evidence for the automatic impact of linguistic stimuli that convey directional information on the motor programming of saccadic eye movements.

In contrast to the influence directional words have on eye movements and attention (e.g., [12], [2]), it is still unclear whether this compatibility effect extends to words that do not directly convey spatial information but do have spatial associations. Estes, Verges and Barsalou [14] first suggested that the processing of linguistic stimuli that do not directly convey spatial information can nevertheless affect visuospatial attention in an involuntary spatial cueing paradigm. In this paradigm, object words referring to entities in the upper or lower visual field (e.g., up = *hat* vs. down = *boot*), were presented at fixation for as short as 100 ms followed by a 50 ms delay, and subsequently a target letter (X or O) was presented in the upper or lower visual field. Target discrimination in compatible locations (e.g. *hat* followed by an upper visual field target) was impeded. These findings were interpreted according to the simulation view of language processing [4]. According to this account, perceptual simulations of the described object engage visual processing resources, subsequently hindering visual target discrimination in compatible locations. Indeed, if visual simulations engage neural mechanisms that are also required for visual perception (e.g. [15]), visual interference effects can be attributed to one part of the visual system being engaged in simulations, and therefore being less efficient for other visual tasks (e.g., visual perception). Similar findings have been reported in sentence-based studies (e.g., [16]) and studies implementing verbs (e.g., *rise*, *fall*) [17]. These interference effects have been interpreted as spatial shifts of attention towards compatible locations, with interference resulting from a perceptual simulation of the word's referent that hinders identification of targets in the same location. Thus, in contrast to typical findings of attentional shifts resulting in facilitated target discrimination (e.g., [2]), the evidence for attentional shifts triggered by words referring to entities with a typical location in the world (e.g., *shoe* vs. *hat*) is indirectly drawn from inhibitory effects.

In summary, evidence regarding facilitation and interference effects of language on visual target detection and discrimination is ambiguous. On the one side, direction words (e.g., *left*, *right*, *up*, *down*) always seem to result in facilitation effects, independent of the participant's task. On the other side, object words seem to influence target processing in an inhibitory manner within visual discrimination paradigms. Here, visual simulations of described events, objects or scenes have been argued to be the cause of the interference effects (e.g., [14]). This might lead to the hypothesis that visual simulations are only activated for object words (e.g., *shoe*, *bird*), and thus impaired discrimination performance in attended locations is only observed when using object words and not direction or location words. Critically, results showing inhibitory effects (e.g., [14], [16]) are only found consistently in discrimination tasks. In detection tasks, either weak facilitation effects or no effects are reported (e.g., [7], [18]). In addition to findings from attentional cueing paradigms, facilitatory effects of object words on motor responses have been reported (e.g. a word such as *bird* or *rise* facilitated upwards responses) (e.g., [18], [19], [20]). Such facilitatory effects are typically discussed within the framework of the experiential-model of language understanding [6]. Hereby words become connected with our experiences when encountering language. For example, we often encounter the word *bird* when someone points to a bird in the sky, and when we are looking upwards to see the bird. Subsequently, these experiential-traces become reactivated whenever we process the word *bird*.

Although various studies have investigated the relationship between language and space in the visuo-motor domain, the functional locus of compatibility effects between language and spatial processing is underspecified. Before being able to draw detailed inferences regarding the mechanisms underlying these effects, clarification regarding which tasks result in automatic spatial compatibility effects for object words is required. Currently, we know that manual responses are typically facilitated and that visual discrimination performance is typically impaired (e.g., [19], [14], respectively). In contrast, directional words result in facilitation effects in various attentional cueing paradigms [2], in manual response tasks as typically implemented in the spatial Stroop paradigm [21], as well as in saccadic response paradigms [12]. Given the importance of gaze direction as a cue for disambiguation processes in spontaneous dialog [22], its influence on creating a shared perspective during dialogue [23], and above all its close coupling with attentional mechanisms (e.g., [24], [25], [26]), it is surprising that no evidence regarding the influence of non-directional words on eye movements has been reported. The current study investigates the influence of object words on saccadic eye movements in the vertical dimension by implementing a paradigm that has previously been used with manual responses [19]. Schwarz and Keus [27], in the context of the SNARC effect (spatial-numerical association of response codes [28]), suggested that manual responses and eye responses should be affected in a similar manner within a SNARC paradigm, if the spatial associations between numbers and space are due to a general association between numbers and space, rather than an overlearned association between low (high) numbers and left (right) hand motor responses (e.g., as the alignment of the number keys on a laptop keyboard). Thus, since the activation of sensory spatial processing during language processing has been suggested to be inevitable for meaning composition processes (e.g., [4], [29]), we expect a general rather than effector-specific relationship between language and space. In accordance with the findings in studies using manual responses (e.g., [18], [19], [20]), we expect that words referring to entities typically located in the upper visual field will facilitate upwards saccades. In contrast, words referring to entities in the lower visual field should facilitate downwards saccades. However, if visual simulations do not only hinder target discrimination but also inhibit eye movements, we should find inhibitory effects.

## Methods

### Participants

18 Students (5 males; age range 20–35 years) participated for monetary reward or course credits. All participants were native German speakers and all of them had normal or corrected-to-normal vision. The experimental procedure was explained to the participants before the experiment and they were briefed about its purpose afterwards.

### Ethics statement

The experimental testing was in agreement with the guidelines for good scientific practice at the University of Tübingen (Germany). This was approved and checked by the Head of Psychology, Faculty of Science, University of Tübingen. Participants' anonymity was always preserved; at no point could the recorded data be associated with a participant's name. All participants provided written informed consent.

### Stimuli

At the beginning of each trial, a centrally presented fixation cross (20 pixels wide and high, corresponding to 0.46° of visual angle), together with two circular response targets (diameter 16 pixels or 0.37°), located 400 pixels (9.20°) above and below the fixation target, were presented. Stimulus words were also presented centrally in 36 pt. Arial in lowercase, but starting with an uppercase letter, which is standard for German nouns. All stimuli were presented in black against a white background. The stimulus word could either be a German noun, or a non-word. All non-words were pronounceable and constructed by taking a German noun (different from the actual ‘up’- and ‘down’-words) and permuting or exchanging some of the letters. Half of the real German nouns denoted objects associated with ‘up’, such as ‘Sonne’ (*sun*) or ‘Vogel’ (*bird*), while the other half was associated with ‘down’, like ‘Schuh’ (*shoe*) and ‘Maus’ (*mouse*) (see Appendix S1). Words were controlled for frequency (http://wortschatz.uni-leipzig.de), length and typical location (vertical axis): For this purpose, 15 volunteers rated 160 nouns on a 5-point Likert-scale. Words selected as ‘down’-words had rating values smaller or equal than 2.2, words selected as ‘up’-words had rating values equal or larger than 3.8. Subsequently, word length and frequency were matched across the two categories of vertical position. This selection process resulted in 39 ‘up’- and 39 ‘down’-words that did not differ significantly with regard to frequency, *t*(76) = 0.39, *p* = .70, or length, *t*(76) = 0.13, *p* = .90, but did differ significantly for the rated position, *t*(76) = 45.67, *p*<.001.

### Apparatus

Stimuli were presented on a 17″ CRT monitor screen (resolution 1280×960). Stimuli were viewed binocularly, at a viewing distance of 61 cm, with the participant's head resting on a chinrest. Stimulus presentation was controlled by custom written software, using the PsychToolbox 3.0 in MATLAB [30], [31], [32]. Eye movements were measured monocularly from the participant's right eye at 1000 Hz with a desktop-mounted Eyelink 1000 system (SR Research, Kanata, Canada), using the Eyelink Toolbox in MATLAB [33].

### Procedure

At the beginning of each block, the Eyelink system was calibrated using its standard nine-point calibration routine. The calibration result was validated with Eyelink's validation routine. Stimulus presentation was not started until the results of both calibration and validation were declared ‘good’ by the Eyelink system.

**Figure 1:**
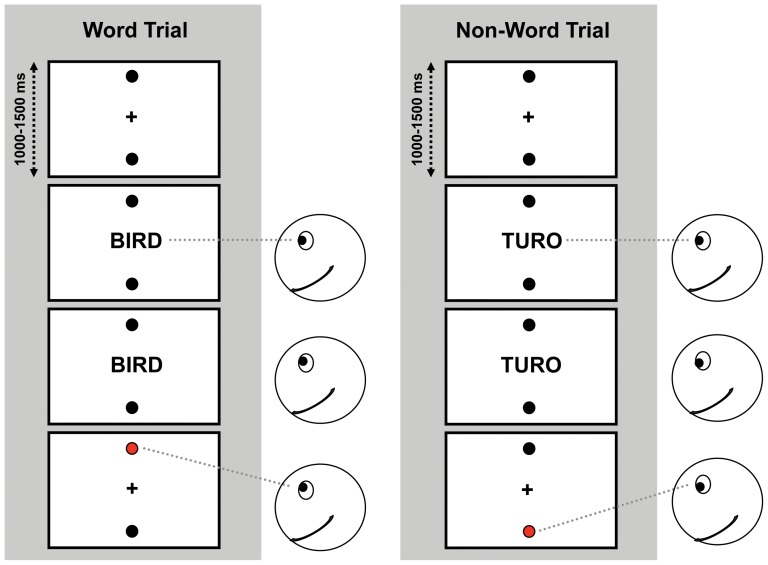
Exemplary trial procedure for a word (left panel) and a non-word trial (right panel). Participants responded in the first half of the experiment to indicate that a word is presented by looking upward (downward) and to indicate that a non-word is presented by looking downward (upward). In the second half of the experiment this mapping was reversed.

Each trial started with the presentation of the fixation cross in the center of the screen and the two horizontally aligned response targets above and below the fixation cross (see Figure 1). After a random delay of 1000 to 1500 ms the fixation cross was replaced by one of the words or one of the non-words. Participants performed a lexical decision task. They were instructed to respond by looking at either the upper or the lower response target. The experiment was run in two blocks, separated by a 5 minute break. In one block, participants were instructed to look at the upper target when the stimulus was a word and at the lower target when it was a non-word. In the other block, the reversed response pattern applied. The order of the two blocks was counterbalanced across participants. Within each block, the 39 ‘up’-nouns, the 39 ‘down’-nouns and the 78 non-words were presented twice in a random order. In both blocks, the same set of stimulus words was used. A practice block of 20 trials from a separate stimulus set was completed before commencing the experimental trials. Consequently, each block contained 332 trials, resulting in a total of 664 trials. The experiment lasted about 45 min.

Saccadic eye movements were detected online, based on a velocity criterion (50°/s). As soon as a vertical saccade was detected, the stimulus word was removed from the display. The instantaneous eye velocity was computed online, by taking the slope of a linear regression of vertical eye position on time over the last 9 samples. After a saccade had been detected, the response target in the direction of the saccade changed color from black to red as soon as eye movement speed dropped below 20°/s, to confirm the response. If no saccade was detected within 3000 ms from stimulus word onset, the trial was aborted and a feedback message (‘Too slow!’) was shown. The next trial started automatically after a 1000 ms interval.

### Data-analysis

**Figure 2:**
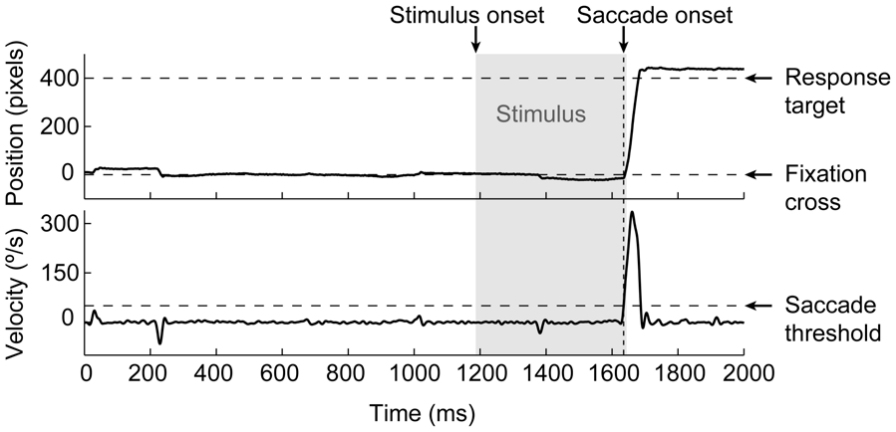
Example of eye movement data. Vertical eye position (upper panel) and eye velocity (lower panel) as a function of time since fixation onset at 0 ms. Saccade onset was defined as the moment where vertical eye movement velocity exceeded the saccade threshold of 50°/s. The shaded area shows the time interval in which the stimulus word was presented.

Eye movement data were analyzed offline in MATLAB to determine the response times (saccadic latencies) more accurately than was possible online and to discard trials with blinks or with saccades which were not made to one of the two response targets. Vertical eye velocity was computed by direct differentiation of eye position with respect to time and then low-pass filtered (3rd order Butterworth filter with 50 Hz cut off). A saccade was considered to be a valid response to the stimulus word if saccade onset (velocity>50°/s) had occurred within 50 ms from stimulus offset and landed within 100 pixels from one of the two response targets. All trials were inspected visually and invalid trials that had passed automatic processing were discarded manually. Figure 2 shows an example eye trace, in which the participant responded with an upward saccade.

Saccadic latencies to real words in which participants responded correctly were analyzed in a 2 (saccade direction) ×2 (stimulus word direction) repeated measures ANOVA with participant as random factor (*F*_1_: by-participants analysis), and in a second ANOVA with stimulus word as random factor (*F*_2_: by-item analysis), using R (version 2.15.2) [34].

## Results

Invalid trials due to blinks, incomplete saccades (not reaching one of the response targets), or multiple saccades were discarded from further analysis. This involved about 15% of the trials with a real word (vs. 18% of the non-word trials). In the remaining real word trials, the response was correct in 92% of the cases. Outliers, defined as saccadic latencies differing more than 2 *SD*s from the mean saccadic latency of a participant in a given condition, were removed from the data set (<5%).

**Figure 3:**
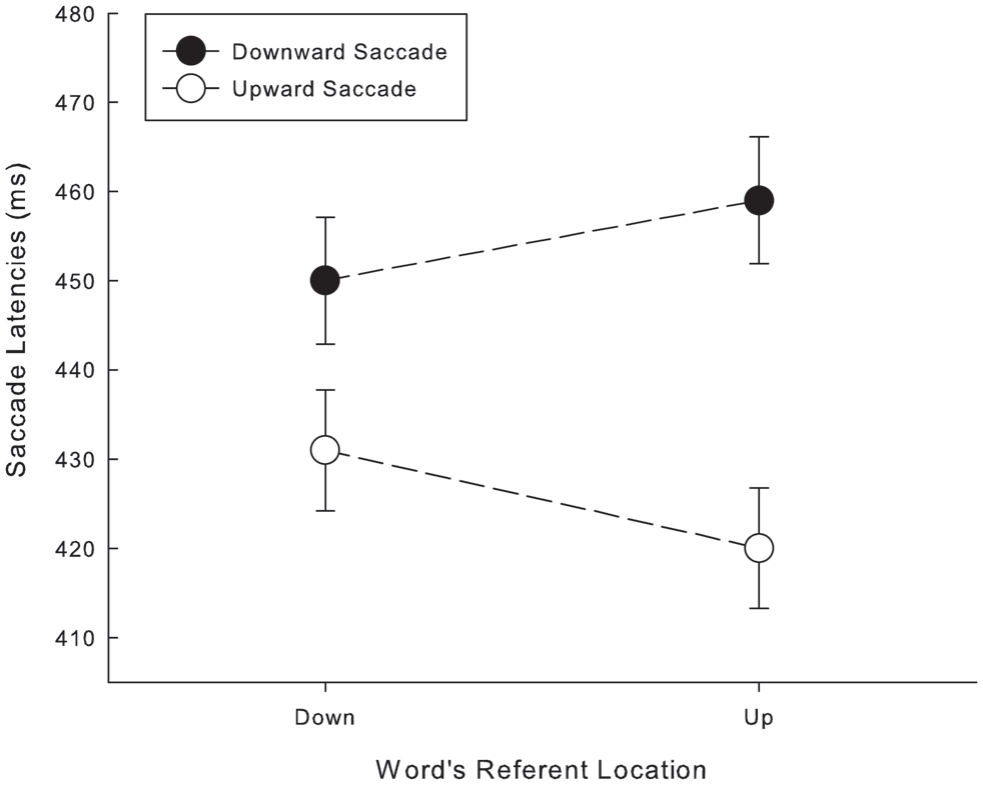
Mean saccadic latencies. Filled circles represent the conditions in which participants responded with a downward eye movement to indicate that the stimulus was an existing German word; open circles show the saccadic latencies when participants responded with an upward eye movement. Error bars represent the 95% confidence intervals of the mean based on within-subject differences [36].

Figure 3 shows the mean saccade latencies for ‘up’- and ‘down’-words in the two response conditions (confidence intervals were calculated according to Loftus and Masson [36]). Saccade latencies in trials with an upward eye movement (*M* = 425 ms) were faster than trials with a downward eye movement (*M* = 454 ms) (*F*_1_ (1,17) = 13.47, *p*<.01; *F*_2_ (1,76) = 202.48, *p*<.001). This is in line with previous findings suggesting significantly faster upwards-directed saccades (e.g., [35]). Saccadic latencies to ‘up’ words did not differ significantly from those for ‘down’ words (*F*_1_ (1,17) = 0.56, *p* = .46; *F*_2_ (1,76) = .008, *p* = .93). Importantly, the interaction between the typical location of the words' referents and saccade direction was significant, *F*_1_ (1,17) = 5.68, *p*<.05; *F*_2_ (1,76) = 15.41, *p*<.001, suggesting that responses were faster when the saccade direction corresponded to the semantic content of the stimulus word. Planned contrasts were calculated (using one-tailed t-tests) in order to analyze whether both upward and downward saccades were facilitated by preceding compatible words. People were faster to launch an upwards saccade in response to a word referring to entities typically encountered in the upper visual world (*M* = 420 ms, *SD* = 48.06) than to words referring to entities typically encountered in the lower visual world (*M* = 431 ms, *SD* = 52.02), *t*_1_(17) = 2.53, *p* = .02, *t*_2_(38) = 1.78, *p* = .04. In contrast, the analysis of downward saccades showed that saccade latencies were faster following down-words (*M* = 450 ms, *SD* = 52.47) than following up-words (*M* = 458 ms, *SD* = 59.78), reflected in a trend for word referent location, *t*_1_ (17) = −1.73, *p* = .05, *t*_2_ (38) = −1.15, *p* = .13.

Due to the use of a novel paradigm measuring saccadic onset latencies in a typical choice response task setup, we additionally performed analyses of log-transformed mean saccadic latencies. The analysis supported the results from the analysis of the non-transformed data. There was a significant main effect of saccade-direction, *F*_1_(,17) = 16.02, *p*<.001, *F*_2_(1,76) = 206.66, *p*<.001. There was no significant difference in saccadic latencies to ‘up’-words and ‘down’-words, *F*_1_(1,17) = 1.29, *p* = .27, *F*_2_(1,76) = .03, *p* = .86. Importantly, there was a significant interaction between word-direction and saccade-direction, *F*_1_(1, 17) = 5.00, *p*<.05, *F*_2_(1,76) = 15.57, *p*<.001.

## Discussion

The current study investigated the impact of words referring to entities in the world (e.g., *sun*, *bird*, *stone*, *shoe*) on saccadic eye movements. In contrast to directional words (e.g., *left*, *right*, *up* and *down*) these words do not directly refer to a location. However, we typically experience these words' referents in a stereotypical location in the world. Zwaan and Madden [6] suggest that language becomes closely connected with our experiences when encountering certain words, and that these experiential traces are reactivated when later processing these words. Indeed, our results show that a wide set of linguistic cues (78 words) results in facilitated eye movements towards a location that is compatible with the referent's typical location in the world. Our findings are in line with previous studies that have shown a direct impact of language on eye-movements (e.g., [9], [12]), emphasizing the strong link between language and visuospatial information processing.

Previous studies investigating the influence of directional words on spatial processing typically implemented visual attention paradigms and reported faster target discrimination or detection in compatible locations (e.g., targets on the left side of a screen are detected faster if cued by the word *left*) [2]. Interestingly, this compatibility effect can also be found when implementing a paradigm requiring eye movements to visual targets on the screen [12]. In contrast to converging evidence suggesting facilitating influence of directional words on target processing in compatible locations, there is mixed evidence regarding the effect of non-directional words on attention. Previous studies using visual discrimination tasks report inhibitory effects, such as impaired discrimination performance in an upper screen location after words such as *bird* [14]. These effects are typically explained by visual simulations of the described event or object in screen locations compatible with the typical location of the word's referent (e.g., [4]). However, these effects seem highly dependent on visual task demands. If the visual target is not an abstract symbol but resembles the described objects (e.g., picture of a bird after the word *bird*), discrimination performance improves (e.g., [37]). In contrast, when using symbolic targets (e.g. letters, squares etc.), visual discrimination is hindered, but simple target detection is facilitated or not affected at all by visual simulations (e.g., [7], [18]). This dissociation between the findings in discrimination and detection tasks might be due to the fact that visual discrimination and detection tasks differ in various aspects, such as the demands for visual processing resources, response selection demands and also, the time course of processing [38]. However, a very recent study suggests that rather than task demands, the usage of multiple word categories in one experiment is a requirement in order to find inhibition effects [39]. Our study uses words from a variety of categories (e.g., living = *bird*, non-living = *sun*, man-made = *hat*, natural = *stone*, etc.). Despite using such a diverse set of words, we do not find interference effects in the latency of saccadic eye movements. Thus, in addition to the usage of multiple word categories, there must be another precondition for the occurrence of interference effects. Most likely, the mechanisms underlying language-space relationships in attentional cueing paradigms and paradigms which require some form of motor response (e.g., eye movements, hand movements) have to be differentiated. Indeed, our paradigm is very similar to previous studies which required manual responses in the vertical dimension, where participants had to decide whether a word referred to something man-made or natural [18], or to perform a lexical decision task [19]. Similar to the current study, these studies have consistently reported facilitating effects. The finding that eye-movements are influenced in a similar fashion to manual responses might suggest a common underlying mechanism [27]. However, what are the mechanisms underlying findings showing the impact of language on spatial task parameters? The following paragraphs will discuss the most likely causes of these compatibility effects.

First, as mentioned previously, it has been suggested that linguistic processing does automatically affect attention. In paradigms implementing attentional cueing tasks, it has been suggested that words result in automatic shifts of attention. These inferences were drawn from both facilitation and inhibition effects (e.g., [14], [7]). These attentional shifts might also underlie the reported facilitation of saccadic eye movements towards compatible locations. In visual attention research it has been suggested that eye movements and attention are closely connected (e.g., [23], [24]). Some models also suggest a common neural network linking attention to the preparation of subsequent saccades to attended locations [25]. Previous studies investigating the effect of words referring to entities with a spatial location typically did not control for eye movements. Thus, it cannot be ruled out with certainty that participants performed saccades to the target location. Therefore, it remains an open question whether actual visual discrimination or looking towards compatible locations is impaired by linguistic cues. Importantly, our study suggests that saccade performance is not hindered by linguistic cues with compatible spatial referents. However, do our findings suggest that words indeed affect visuospatial attention? As mentioned above, converging evidence suggests that saccadic eye movements are typically preceded by shifts in attention (e.g., [40]). Also, in everyday life attention and eye movements are closely connected, and we typically move our eyes to previously attended locations that are judged to be worth making a saccade towards [2]. However, in our paradigm, participants did not choose which location was worth looking at, but were required to move their eyes in order to respond towards a specific target at a specific location. Thus, it remains open whether these findings support an attention account or, in contrast, have to be attributed to other cognitive processes, for example, to facilitation in response selection stages. Future studies should assess whether linguistic cues do indeed automatically affect attention across various experimental paradigms, and whether internal (covert) and external attention (overt) [41], such as eye movements, are affected in qualitatively different ways.

Interestingly, as mentioned above, the experimental setup in the current study is very similar to previous studies using manual responses (e.g., [18], [19]). This study extends these previous findings that words reactivate experiential traces on a motor level (e.g., [5], [18], [19]) by showing that this reactivation of experiential traces is not limited to motor aspects of arm movement planning, but can also be observed if the task requires the planning and performing of saccadic eye movements. According to Schwarz and Keus, similar findings across two response effectors (e.g., hand vs. eye) suggest that response selection stages are the locus of the effect [25]. Yet, why would it be easier to select an upward response after words such as *airplane*? On the one hand, experiences and frequency counts throughout the lifetime might be responsible for preferring the selection of an upwards response following the word *airplane*. Also, prototype-based categories (e.g., *bird* belongs to the category “flying animal”) might become activated by the words, that subsequently result in upward or downward response activations. Additionally, according to the theory of event-coding (TEC), these words might automatically activate the spatial features ‘up’ or ‘down’, and if the same features are required for responding, these pre-activations of spatial features may result in facilitation effects if the features are indeed available at the time-point of responding [42]. Taken together, despite good arguments to suggest that response selection stages might be influenced by linguistic cues, future studies are needed to investigate the mechanisms underlying this effect.

In what way do our findings extend previous studies investigating the relation between language and eye-movements? In studies using the visual world paradigm (e.g., [9]), participants have to simultaneously process language and view visual scenes or arrays of objects. Here, when processing sentences such as “*He eats the* …” participants typically restrict the domain of the upcoming word to something edible, and as a result, preferably focus on edible objects on the screen. In contrast, our experiment did not allow participants to search the visual field for targets related to the linguistic information (for discussion of the visual world paradigm see also [43]). Our participants simply had to move their eyes upwards or downwards according to whether a word or non-word was presented. Thus, facilitated eye movements in our experiments suggest that a wide range of linguistic stimuli have a direct impact on saccadic eye movements, even if the visual arrays are non-informative. These findings might also be interesting from a communicative perspective of language processing. Richardson, Dale and Kirkham showed that a very close coupling of eye movements between speakers takes place during spontaneous dialogue [22]. We often speak, point and gesture at the same time in order to share information about our external world. Our study shows that even when there is no reason to attend to one location over another (and no gesturing or pointing involved) words automatically have an impact on subsequent eye movements, and consequently might help in routinely creating a shared perspective during dialogue.

In summary, we have demonstrated that a wide set of words referring to entities with a typical spatial location in the world facilitates saccadic eye movements towards compatible locations on the screen, extending previous results using only direction words (e.g., *left*, *right*, *up*, *down*). These results have important implications for the embodied view of language processing; words can affect subsequent eye movement performance in a similar way as they influence manual responses [6]. These findings suggesting a direct link between language and saccadic eye-movements can also provide a further step towards understanding the close coupling between language and visual information processing, and can shed light on how natural communication results in creating a shared perspective (e.g., [22], [23], [44]).

## Supporting Information

Appendix S1 **Stimulus material.**
(PDF)
