# Supplementary material for: Reading “Sun” and Looking Up: The Influence of Language on Saccadic Eye Movements in the Vertical Dimension
Source: PLoS One. 2013 Feb 27;8(2):e56872. doi: 10.1371/journal.pone.0056872 (PMC3584096; doi:10.1371/journal.pone.0056872)
Supplement: Appendix S1 — Stimulus material. (PDF) [file pone.0056872.s001.pdf]

Supporting Information

Materials

German Noun	Location	Length	Freq. Class	Frequency	*M* Rating	*SD* Rating	Min	Max
**Tiefe**	down	5	11	3084	1,00	0,00	1	1
**U-Bahn**	down	5	11	2244	1,07	0,26	1	2
**Abgrund**	down	7	13	742	1,07	0,26	1	2
**Grab**	down	4	11	1737	1,13	0,35	1	2
**Gruft**	down	5	15	116	1,13	0,35	1	2
**Keller**	down	6	10	5317	1,13	0,35	1	2
**U-boot**	down	5	13	477	1,13	0,35	1	2
**Unterwelt**	down	9	14	328	1,13	0,35	1	2
**Maulwurf**	down	8	15	128	1,20	0,41	1	2
**Untergrund**	down	10	12	1106	1,20	0,56	1	3
**Höhle**	down	5	13	602	1,21	0,43	1	2
**Katakombe**	down	9	18	16	1,23	0,44	1	2
**Wurzel**	down	6	13	474	1,27	0,59	1	3
**Fußboden**	down	8	14	380	1,40	0,51	1	2
**Fußsohle**	down	8	17	32	1,40	0,51	1	2
**Sohle**	down	5	15	175	1,40	0,51	1	2
**Wurm**	down	4	13	599	1,40	0,51	1	2
**Taucher**	down	7	13	744	1,47	0,64	1	3
**Graben**	down	6	12	1052	1,60	0,51	1	2
**Gras**	down	4	12	1135	1,60	0,51	1	2
**Schlucht**	down	8	14	384	1,60	0,83	1	4
**Boden**	down	5	8	14759	1,67	0,82	1	3
**Erdreich**	down	8	14	369	1,67	0,62	1	3
**Flussbett**	down	9	16	91	1,67	0,72	1	3
**Fuß**	down	3	9	8262	1,67	0,72	1	3
**Stein**	down	5	10	4293	1,67	0,62	1	2
**Sumpf**	down	5	13	413	1,67	0,49	1	2
**Tümpel**	down	6	15	117	1,67	0,49	1	2
**Pfütze**	down	6	15	114	1,73	0,59	1	3
**Schienen**	down	8	12	1143	1,73	0,70	1	3
**Teppich**	down	7	11	1961	1,73	0,80	1	3
**Tunnel**	down	6	11	2239	1,73	0,70	1	3
**Maus**	down	4	12	1264	1,87	0,52	1	3
**Schotter**	down	8	15	156	1,87	0,52	1	3
**Fluss**	down	5	11	2413	1,93	0,70	1	3
**Klee**	down	4	14	293	2,07	0,80	1	3
**Gehweg**	down	6	13	626	2,13	0,64	1	3
**Erde**	down	4	9	9658	2,14	0,86	1	3
**Straße**	down	6	7	27894	2,20	0,77	1	3
**Krone**	up	5	12	1110	4,07	0,46	4	5
**Höhepunkt**	up	9	10	6144	4,14	0,86	2	5

**Decke**	up	5	11	2085	4,20	0,77	3	5
**Hochland**	up	8	14	215	4,20	0,56	3	5
**Giebel**	up	6	15	158	4,23	0,73	3	5
**Burg**	up	4	12	1375	4,27	0,46	4	5
**Empore**	up	6	15	199	4,29	0,47	4	5
**Hochebene**	up	9	16	67	4,40	0,63	3	5
**Dachbalken**	up	10	17	27	4,53	0,52	4	5
**Spitze**	up	6	8	14518	4,57	0,76	3	5
**Planet**	up	6	12	963	4,60	0,63	3	5
**Turm**	up	4	11	1857	4,60	0,51	4	5
**Vogel**	up	5	11	2839	4,60	0,51	4	5
**Hochsitz**	up	8	17	43	4,67	0,49	4	5
**Drachen**	up	7	13	434	4,73	0,46	4	5
**Höhe**	up	4	8	26230	4,73	0,46	4	5
**Vogelnest**	up	9	18	21	4,73	0,46	4	5
**Ballon**	up	6	14	290	4,80	0,41	4	5
**Falke**	up	5	14	224	4,80	0,41	4	5
**Hochseil**	up	8	17	42	4,80	0,41	4	5
**Nest**	up	4	13	536	4,80	0,41	4	5
**Wolke**	up	5	13	744	4,80	0,41	4	5
**Dach**	up	4	9	8002	4,87	0,35	4	5
**Flugzeug**	up	8	10	5163	4,87	0,52	3	5
**Gebirge**	up	7	14	370	4,87	0,35	4	5
**Hochhaus**	up	8	13	658	4,87	0,35	4	5
**Mond**	up	4	11	2029	4,87	0,35	4	5
**Stern**	up	5	10	4796	4,87	0,35	4	5
**Berg**	up	4	10	5288	4,87	0,35	4	5
**Adler**	up	5	11	1827	4,93	0,26	4	5
**Alpen**	up	5	11	2176	4,93	0,26	4	5
**Gipfel**	up	6	10	4384	4,93	0,26	4	5
**Komet**	up	5	15	185	4,93	0,26	4	5
**Zepellin**	up	8	21	1	4,93	0,26	4	5
**Himmel**	up	6	9	7752	5,00	0,00	5	5
**Satellit**	up	8	12	958	5,00	0,00	5	5
**Sonne**	up	5	9	9970	5,00	0,00	5	5
**Ufo**	up	3	16	92	5,00	0,00	5	5
**Weltall**	up	7	13	699	5,00	0,00	5	5
